# EEG Signal Analysis for Diagnosing Neurological Disorders Using Discrete Wavelet Transform and Intelligent Techniques †

**DOI:** 10.3390/s20092505

**Published:** 2020-04-28

**Authors:** Fahd A. Alturki, Khalil AlSharabi, Akram M. Abdurraqeeb, Majid Aljalal

**Affiliations:** Electrical Engineering Department, College of Engineering, King Saud University, Riyadh 800-11421, Saudi Arabia; Falturki@ksu.edu.sa (F.A.A.); encom.akram@gmail.com (A.M.A.); algalalmajid@hotmail.com (M.A.)

**Keywords:** artificial neural network, autism spectrum disorder, band power, discrete wavelet transform, electroencephalogram, entropy, epilepsy, k-nearest neighbor, linear discriminant analysis, support vector machine

## Abstract

Analysis of electroencephalogram (EEG) signals is essential because it is an efficient method to diagnose neurological brain disorders. In this work, a single system is developed to diagnose one or two neurological diseases at the same time (two-class mode and three-class mode). For this purpose, different EEG feature-extraction and classification techniques are investigated to aid in the accurate diagnosis of neurological brain disorders: epilepsy and autism spectrum disorder (ASD). Two different modes, single-channel and multi-channel, of EEG signals are analyzed for epilepsy and ASD. The independent components analysis (ICA) technique is used to remove the artifacts from EEG dataset. Then, the EEG dataset is segmented and filtered to remove noise and interference using an elliptic band-pass filter. Next, the EEG signal features are extracted from the filtered signal using a discrete wavelet transform (DWT) to decompose the filtered signal to its sub-bands delta, theta, alpha, beta and gamma. Subsequently, five statistical methods are used to extract features from the EEG sub-bands: the logarithmic band power (LBP), standard deviation, variance, kurtosis, and Shannon entropy (SE). Further, the features are fed into four different classifiers, linear discriminant analysis (LDA), support vector machine (SVM), k-nearest neighbor (KNN), and artificial neural networks (ANNs), to classify the features corresponding to their classes. The combination of DWT with SE and LBP produces the highest accuracy among all the classifiers. The overall classification accuracy approaches 99.9% using SVM and 97% using ANN for the three-class single-channel and multi-channel modes, respectively.

## 1. Introduction

The electroencephalogram (EEG) signals reflect the electrical activities of brain behaviors. The signal-processing techniques based on EEG signals analysis form an important clinical tool for monitoring and diagnosing neurological brain disorders such as autism spectrum disorder (ASD) and epilepsy disorders because they reflect the electrical activities or disorders of neurons in the human brain. Brain disorders, such as ASD and epilepsy disorders, are defined by such activities in the human brain. Currently, most brain disorder diagnoses are performed manually by neurologists or skilled clinicians through visual inspection of EEG signals. The human brain is the most complex part of the human body and provides a wide variety of information related to limbic movements and neurological disorders. In recent years, researchers in multidisciplinary fields of engineering, neuroscience, microelectronics, bioengineering, and neurophysiology have attempted to take advantage of the information provided by EEG signals for several application domains, such as controls, communications, and medical diagnosis. Currently, several types of studies are being conducted in this field to build and improve an efficient diagnosis system.

Epilepsy is among the most common brain disorders, affecting approximately 65 million people worldwide [1]. Electroencephalography is the most useful diagnostic tool for epilepsy [2]. Several studies have focused on building computer-aided diagnoses for epilepsy. For example, Nigam and Graupe [3] proposed an EEG-based computer-aided diagnosis for epilepsy using a multistage nonlinear preprocessing filter combined with an artificial neural network (ANN). Their proposed technique achieved an accuracy of 97.2%. Kannathal et al. [4] compared different entropy algorithms and proposed the use of entropy values to distinguish neurotypical EEGs from epileptic EEGs. They used an adaptive neuro-fuzzy inference system for classification and achieved an accuracy of 92.2%. Alternatively, Sadati et al. [5] used an adaptive neural fuzzy network for an epilepsy diagnosis. Features were extracted using the energy of the discrete wavelet transform (DWT) sub-bands. However, their proposed method achieved an accuracy of approximately 85.9%. Ocak [6] proposed a method employing approximated entropy for feature extraction with DWT and achieved an accuracy of over 96% when the DWT was employed and 73% in its absence. Instead of classifying only sets A and E, Nunes et al. [7] considered the whole dataset (Sets A, B, C, D, E) provided by Bonn University and investigated several combinations of feature extraction and classification methods. It was noted that the best performance was achieved using the wavelet coefficients as feature extractors and optimum path forest as a classifier to achieve an 89.2% average accuracy. Subasi et al. [8] investigated different analysis techniques to reduce the dimension of EEG data and combined the EEG data with principal component analysis (PCA), linear discriminant analysis (LDA), and independent component analysis (ICA). The wavelet transform was used by Subasi [9] for feature extraction and an expert model for classification. This proposed technique achieved an overall accuracy of 94.5%. Recently, Chen [10] presented a dual-tree complex wavelet transform-Fourier as a feature-extraction method and used the nearest neighbor classifier for classification, and the proposed method achieved a perfect classification accuracy (100%). Another recent method that also achieved a perfect classification rate was proposed by Djemili et al. [11] using empirical mode decomposition for feature extraction and a multilayer perceptron neural network as a classifier.

Further, other researchers have attempted to improve a computer-aided diagnosis system for ASD based on EEG signal analysis [12]. Several previous researches have investigated computer-aided diagnosis (CAD) techniques to diagnose ASD. In the work presented by A. Sheikhani et al. [13], they used the short-time Fourier transform (STFT) technique to extract the features of the EEG signal and then used k-nearest neighbors (KNN) as a classifier. The overall accuracy achieved by this method is up to 82.4%. In a later paper [14], they further developed the proposed method using larger data for testing to obtain an accuracy of 96.4%. Ahmadlou et al. [15] discussed the fractal dimension (FD) for measuring the dynamic changes and complexity in the brains of those diagnosed with ASD. A radial basis function was used as a classifier. This method achieved an accuracy of up to 90%. In another study, the same authors also introduced ASD diagnosis using a visibility graph (VG) technique [16], fuzzy synchronization likelihood (Fuzzy SL), and an enhanced probabilistic neural network classifier [17]. Both proposed methods obtained an accuracy of around 95.5%. Bosl [18] used the minimum mean-square error as a feature vector of the EEG signals, and multiclass KNN, naive Bayesian, and support vector machine (SVM) were used as classification algorithms to classify signals from neurotypical and autistic individuals. The classification accuracy was over 80% for children aged nine months, close to 100% for boys aged nine months, and between 70% and 90% for children between the ages of twelve and eighteen months. For girls, the overall classification accuracy was the highest at an age of six months. In the work of Alhaddad et al. [19], they used optimum preprocessing techniques in their study and employed two feature-extraction techniques: time and frequency domains (raw data and Fast Fourier Transform (FFT)). In the classification, they used a fisher linear discriminant as a classifier and obtained an overall classification accuracy up to 90%. In another study, the same dataset and processing techniques used by Alhaddad were used by Alsaggaf et al. [20] to diagnose ASD, but they did not employ filtering techniques and obtained an accuracy of 80.27%. Fan et al. [21] presented spectral features of EEG signals in conjunction with therapist ratings of behavioral connections, enjoyment, discouragement, boredom, and difficulty to train a group of classification models. They used seven classification techniques, namely, Bayes network, naive Bayes, multilayer perceptron, SVM, KNN, decision tree classifier (J48), and random forest, and compared the results thereof to obtain an overall classification accuracy between 75% and 85%. In our recent work [22], we used two types of neurological brain disorders dataset epilepsy and autism disorders and studied the effect of multi-channel processing on the diagnosis accuracy. DWT was combined with SD, kurtosis and LPB as a feature-extraction techniques and SVM technique is used as a classifier. We obtained an overall classification accuracy up to 98% and 96.5% for epilepsy and autism, respectively.

In the present study, we have developed a single system that diagnoses neurological brain disorders in two modes with high accuracies. The first mode is the diagnosis of two classes: epilepsy versus Neurotypical and ASD versus Neurotypical. The second mode, which is the most important contribution to this study, is the diagnosis of three classes: epilepsy versus ASD versus neurotypical. Different feature extraction and EEG classification techniques are investigated to assist neurologists in the diagnosis of those neurological brain disorders. After applying the preprocessing technique, the most important method for feature extraction, DWT, is used. With this method, we have combined several techniques—logarithmic band power (LBP), standard deviation (SD), variance, kurtosis, and Shannon entropy (SE)—and used four types of classifiers—LDA, SVM, KNN, and ANNs—for our investigations.

The remainder of this work is arranged as follows. Section 2 describes the EEG data used description and EEG signal preprocessing and processing methods, including filtering, feature extraction, and classification techniques. Section 3 presents the results and discussion. Finally, Section 4 presents the conclusion and future work.

## 2. Methods

**Figure 1:**
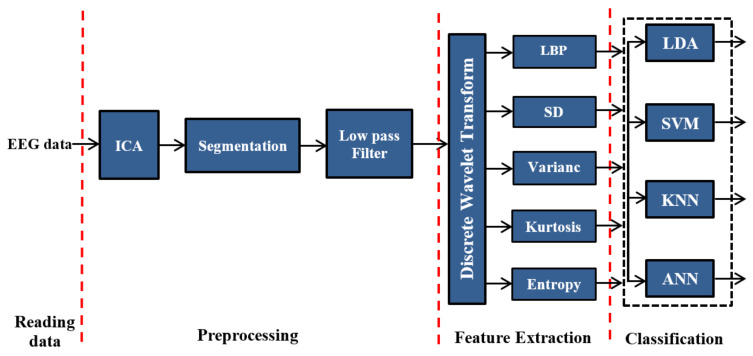
Block diagram of the proposed approaches based on discrete wavelet transform (DWT).

In this section, the proposed feature-extraction and classification techniques are described, as well as their validation using MATLAB software tools (MathWorks, Natick, MA, USA). The block diagram of the proposed method is shown in Figure 1, which highlights the proposed methods based on DWT. In this diagram, the EEG data are first read and then the eye artifacts have been removed from the recorded signals by ICA technique. Then, after artifacts removing process, the EEG signals will be segmented into fixed time windows of 50 s. Next, we feed the output of the segmentation process into a band-pass filter to remove the noises. To extract the features of EEG signals, construct the feature vectors, and improve classification accuracy, LBP, SD, variance, kurtosis, and entropy are used along with DWT. 

In the proposed system, linear discriminant analysis LDA, SVM, KNN, and ANN techniques were applied as classifiers. We implemented and verified all possible combinations of the proposed methods.

### 2.1. Dataset Descriptions

Three types of datasets have been used for implementing and verifying our methods. The first two datasets are used for epilepsy diagnosis, and the third is used for autism diagnosis. The first dataset is supplied by the Bonn University, Germany, and included five sets, named A, B, C, D, and E. Each set contains exactly one hundred single-channel EEG signals. Sets A and B were collected from scalp EEGs of neurotypical persons, whereas sets C, D, and E were collected using intracranial EEGs from epileptic persons. The total period of each signal is approximately 23.6 s. The data were collected with a sampling frequency of 173.61 Hz. The reference provided in [23] shows a more detailed description of this dataset. The research team from MIT, USA [24], provides the second dataset, which includes 906 h of EEG data collected from 23 epileptic patients. In this study, only data for the first twelve epileptic subjects were used, along with those of eleven neurotypical subjects. This data includes 23 EEG channels with a sampling frequency of 256 Hz [25].

The third dataset was supplied by King Abdulaziz University (KAU) Brain–Computer Interface (BCI) Group, Jeddah, Saudi Arabia. The dataset was collected in a relaxed state and split into two groups: the first group was named the neurotypical group and included data from ten healthy volunteer subjects (all males, age 9–16 years) with typical intelligence and without any mental disorders. The second group was labeled the autistic group and included nine subjects (six males and three females, aged 10–16 years) with ASD. The EEG signals were collected from the subjects’ scalps in a relaxed state using an EEG data-acquisition system that included the following components: a g.tec EEG cap with high accuracy, 16 Ag/AgCl sensors (electrodes) based on the 10–20 international acquisition system, g.tec USB amplifiers (gtec medical engineering company, Schiedlberg, Austria), and BCI2000 software (The Brain-Computer Interface R&D Program at the Wadsworth Center of the New York State Department of Health in Albany, NY, USA). The dataset was filtered by a band-pass filter with a passband of 0.1–60 Hz, and a notch filter was used with a stopband frequency of 60 Hz. All EEG signals were digitized at a sampling frequency of 256 Hz. The EEG collection time ranged from 12 to 40 min for autistic patients with a total of up to 173 min. For neurotypical patients, the recording varied between 5 and 27 min with a total of up to 148 min.

### 2.2. Preprocessing

In the EEG signals acquisition stage, the EEG data were recorded with the artifacts, noises and interferences from different sources such as the magnetic field of electronic devices, mobiles wave, power-line, blood pressure, breathing, limb movement, or other human parties [26,27]. In this study, the first step in the preprocessing stage is to apply the ICA technique and adaptive filtering in order to remove the eye artifacts [28]. The EEG signals that were recorded from four electrodes around the eyes are used as reference signals to remove eye blinks artifacts. Then, the EEG dataset is split into an equal segments with a specific length to ensure that the amount of information is equal in each segment. In the present study, 50 s is selected as a segment length because it produced better experimental results. After the EEG signals were segmented, the EEG segments were filtered to remove the noises and interferences generated during EEG signal recording. The filtering technique aims to remove all the noise and interference, enhancing the signal to noise ratio to improve and increase the classification accuracy results. In this work, different filtering methods were used such as finite impulse response (FIR) filters (Equiripple, Kaiserwin, etc.) and infinite impulse response (IIR) filters (Chebyshev I, Chebyshev II, Butterworth, Elliptic, etc.). However, Elliptic band pass (0.1–60 Hz) provides better experimental results compared with the other types of filters because the implementation of the elliptic filter requires less memory, less calculation and provides reduced delay time. 

### 2.3. Feature Extraction

The feature-extraction method is important for EEG signal processing to achieve the best possible performance. The EEG signals are recorded and segmented into long time-series, which is necessary for working with a very small number of values that describe the characteristics of the EEG signal. These values are called features and are aggregated into a vector named the feature vector. Thus, the feature-extraction methods are defined as the techniques that transform signals into a feature vector. There are several types of feature-extraction techniques used to extract features. In the present work, the most popular and widespread technology has been used, namely, DWT. In this study, we propose the use of DWT based on LBP, SD, variance, kurtosis, and entropy to form the feature vectors.

The STFT is unsuitable for analyzing non-stationary signals, such as EEG. This is because STFT offers a constant resolution at all frequencies. To analyze different frequencies with different resolutions, the wavelet transform technique, which uses multi-resolution, is employed. In addition, the wavelet transform can offer a smaller number of features for the signal to be processed; this implies that it may be suitable for avoiding the associated dimensionality problem. Thus, wavelet transforms analyze the characteristics of the signal in the time and frequency domains by decomposing such signals into several functions using a single function [29]. This function is called the mother function and is given by

(1) $$\psi (t)=\frac{1}{\sqrt{2}}\psi (\frac{t-y}{x})\ldots x,y\in S,x>0,$$

where *x* and *y* are the scaling and shifting parameter, respectively, and *S* is the wavelet space. The following equation shows the wavelet transform.

(2) $$F(x,y)=\frac{1}{\sqrt{x}}\int_{S}\psi (\frac{t-y}{x})dt$$

In this work, we employed DWT because it provides a highly efficient wavelet representation. In first-level decomposition, low- and high-pass filters are frequently employed for obtaining the representation of the digital signal as an approximation (A1) and detail (D1) coefficients. The equation that defines DWT decomposition is as follows:

(3) $$f\left(t\right)=\overset{k=+\infty }{\underset{k=-\infty }{\sum }}C_{n,k}\emptyset \left(2^{-n}t-k\right)+\overset{k=+\infty }{\underset{k=-\infty }{\sum }}\overset{k=+\infty }{\underset{k=-\infty }{\sum }}2^{\frac{-j}{2}}d_{j,k}\psi \left(2^{-j}t-k\right)$$

where $\;d_{j,k}$ and $C_{j,k}$ represent the approximation and detail coefficients, respectively, *n* is the level and *∅* is the function of scale. The first approximation is decomposed, and the process is repeated again. At the end of the process, the number of decomposed signals is *n*+1. In this study, we employed Daubechies 4 (db4) as the mother wavelet function; level 4 is selected because it provides the best characteristics for signal features that are classified successfully.

**Figure 2:**
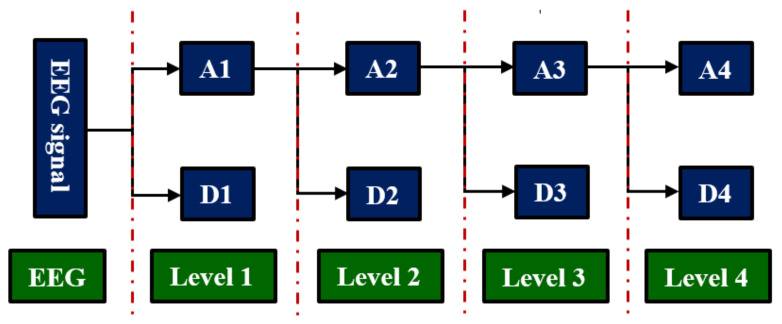
Electroencephalogram signal decomposition through 4-level DWT.

The DWT technique decomposes a filtered signal into approximation (A1: 0.1–30 Hz) and detail (D1: 30–60 Hz) coefficients to obtain the first level of decomposition. The approximation coefficients in the first level are further decomposed into the approximation (A2: 0.1–15 Hz) and detail (D2: 15–30 Hz) coefficients in the second level. In the third level, the approximation coefficients in the second level are decomposed into the approximation (A3: 0.1–8 Hz) and detail (D3: 8–15 Hz) coefficients. Finally, the approximation coefficients in the third level are further decomposed into the approximation (A4: 0.1–4 Hz) and detail (D4: 4–8 Hz) coefficients in the fourth level. Finally, coefficients D1–D4 and A4 are obtained as shown in Figure 2 [22]. After obtaining all detail coefficients in every level (D1, D2, D3 and D4) and approximation coefficients in last level (A4), different combinations of these coefficients were tested to obtain the best result. However, the highest overall classification accuracy was achieved using all of them. 

$S\left(n\right)$ is a discrete signal, where n=1,2,…, N, and *N* is the number of signal samples; the feature vectors were formed using the following techniques:

The variance of the signal

(4) $$V_{s}=\frac{1}{N}\overset{N}{\underset{n=1}{\sum }}{\left(S\left(n\right)-\mu_{s}\right)}^{2}$$

where $\mu_{s}$ is the mean of signal samples [30].

The SD of the signal

(5) $$\sigma_{s}=\sqrt{\frac{1}{N}\overset{N}{\underset{n=1}{\sum }}{\left(S\left(n\right)-\mu_{s}\right)}^{2}}$$

where $\mu_{s}$ is the mean of signal samples [31].

The kurtosis of the signal

(6) $$kurt=E\left[{\left(\frac{S\left(n\right)-\mu_{s}}{\sigma_{s}}\right)}^{4}\right]$$

where $E\left[\;\right]$ is the expected value, $\mu_{s}$ is the mean, and $\sigma_{s}$ are the mean and standard deviation of signal samples [32].

The non-normalized SE [33]

(7) $$Ent=\overset{N}{\underset{n=1}{\sum }}{\left|S\left(n\right)\right|}^{2}log{\left|S\left(n\right)\right|}^{2}$$

The LBP of the signal [34]

(8) $$LBP=\log(\frac{1}{N}\overset{N}{\underset{n=1}{\sum }}{\left|S\left(n\right)\right|}^{2}$$

### 2.4. Classification and Cross-Validation

#### 2.4.1. LDA and SVM Techniques

Both LDA and SVM classification techniques use hyperplane separation to classify their inputs. LDA is a generalization of Fisher’s linear discriminant and is dependent on the covariance matrices and mean vectors of the feature vectors for individual classes. LDA also uses a hyperplane to differentiate between classes, reducing the variance within the class and exploiting the variance between the classes [35]. The SVM classifier is a supervised learning approach that analyzes data and distinguishes patterns and is used for classification and regression analysis. Given a set of training examples, an SVM training algorithm builds a model (i.e., the separation hyperplane) that assigns new examples into single categories [36]. In the present study, we used a linear SVM because a non-linear SVM is expected to have higher computational costs and longer computation time. In essence, the SVM is a binary (two-class) classifier. To address the three-class classification problem, in this study, we combine three SVM classifiers using a one-versus-all method.

#### 2.4.2. KNN and ANN Techniques

The KNN classifier is the simplest machine-learning algorithm and distinguishes objects by a majority vote of its *k*-nearest neighbors [37]. In the current work, *k* is selected to equal five for all experiments. We have also used ANN as a classifier, which is an information-processing system that simulates the process of human cognition. During the training process, the feature vectors are applied to the network to adjust its variable parameters, weights, and biases. Thus, the relationships between input and output patterns were captured. In the present work, an ANN system has been designed with one layer in each section—the input, hidden, and output layers—using MATLAB. The hidden layers have been designed with five nodes and the output layer with a number of nodes equal to the number of output classes. In contrast, the number of nodes in the input layer depends on the number of obtained features. 

**Figure 3:**
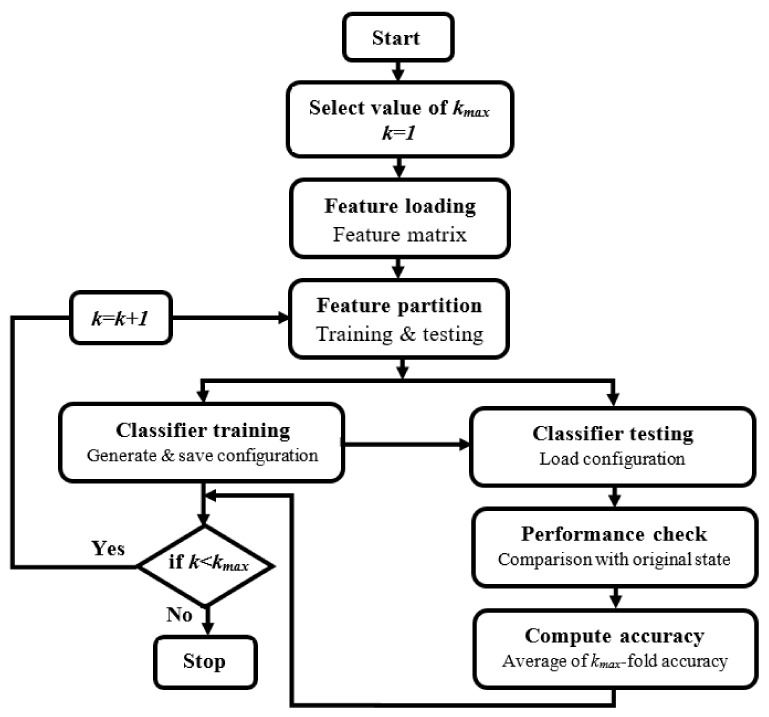
Methodology for k-fold cross-validation.

In this technique, the dataset is arbitrarily separated into *k* equal parts (subsets) [38]. All the subsets are employed for training except for one used in the testing phase (for validation). This procedure was repeated *k* times (*k*-fold), where each subset is used once for testing. In this experiment, we used 10-fold cross-validation, where all the EEG signal features were loaded from the feature vector that was extracted from the feature-extraction section and transmitted to the cross-validation section. Next, these features were split into a 90% subset for training and a 10% subset for testing. Each time, a vector was transmitted into the testing classifier. Then, the cross-validation algorithm compared the result of the testing classifier with the state of the original test features for validation. This process was repeated ten times; each time, one vector was transmitted into the testing classifier. Finally, the results were averaged to produce a single overall classification accuracy. Figure 3 shows the cross-validation methodology for 10-fold cross-validation.

(9) $$accuracy=\frac{1}{k_{max}}\overset{k_{max}}{\underset{k=1}{\sum }}\left[\frac{M{\left(k\right)}_{correct}}{M_{total}}\right]*100\%$$

where $M_{total}$ is the total number of vectors to be classified, $M{\left(k\right)}_{correct}$ is the number of correct vectors in k iteration, and $k_{max}$ is the number of folds.

## 3. Results and Discussion

As mentioned earlier, what distinguishes this study from previous studies is that we worked to develop a single system that diagnoses neurological disorders in two modes with high accuracies. The first mode is the diagnosis of two classes: epilepsy versus Neurotypical and ASD versus Neurotypical. The second mode, which is the most important contribution to this study, is the diagnosis of three classes: epilepsy versus ASD versus Neurotypical. For this purpose, the ICA technique is used to remove the artifacts from the raw signals. Then, the EEG signals were segmented into fixed time windows and then filtered using a band-pass elliptic filter with a frequency band of 0.1–60 Hz. Next, the features were extracted from the EEG signal frequency bands; delta, theta, alpha, beta, and gamma using DWT combined with the LBP, SD, variance, kurtosis, and entropy methods. Finally, four types of classifiers are employed—LDA, SVM, KNN, and ANNs—for our investigations. In the following subsections, the classification accuracy results of the proposed methods will be presented, analyzed, and discussed for both two-class and three-class and both single-channel mode and multi-channel mode diagnosis using three types of EEG datasets: Bonn university dataset, CHB-MIT dataset and King Abdulaziz University dataset.

### 3.1. Two-Class Classification

We began with two types of neurological brain disorders: epilepsy and ASD. The comparisons of EEGs for epileptic and neurotypical patients and those of autistic and neurotypical patients are presented separately. In our work, single-channel and multi-channel modes were studied, and in our proposed system, several feature-extraction methods were combined with several classification methods. 

**Figure 4:**
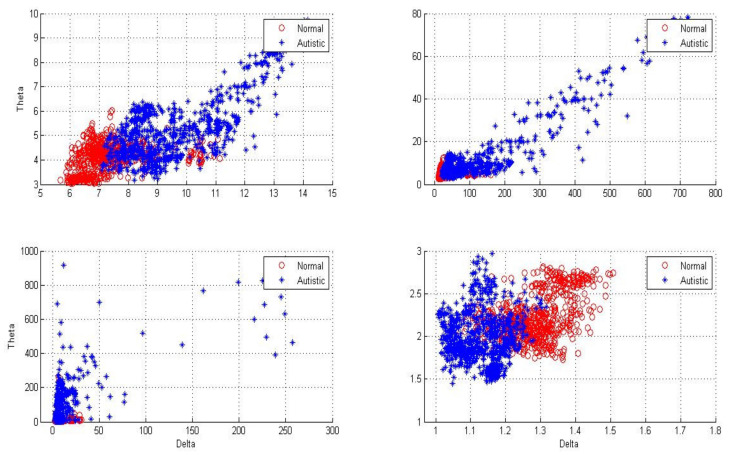
Two-dimensional plot of feature vectors of autistic and neurotypical data using (**A**) DWT + logarithmic band power (LBP), (**B**) DWT + standard deviation, (**C**) DWT + kurtosis, and **(D**) DWT + entropy.

**Figure 5:**
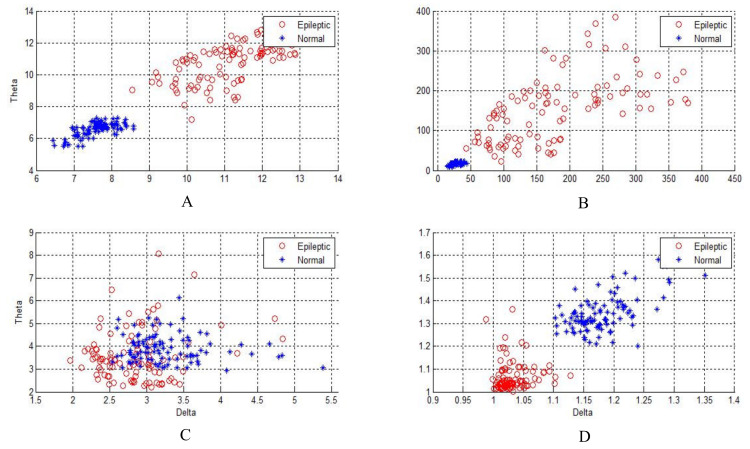
Two-dimensional plot of feature vectors of epileptic and neurotypical data using (**A**) DWT + LBP, (**B**) DWT + standard deviation, (**C**) DWT + kurtosis, and (**D**) DWT + entropy.

**Figure 6:**
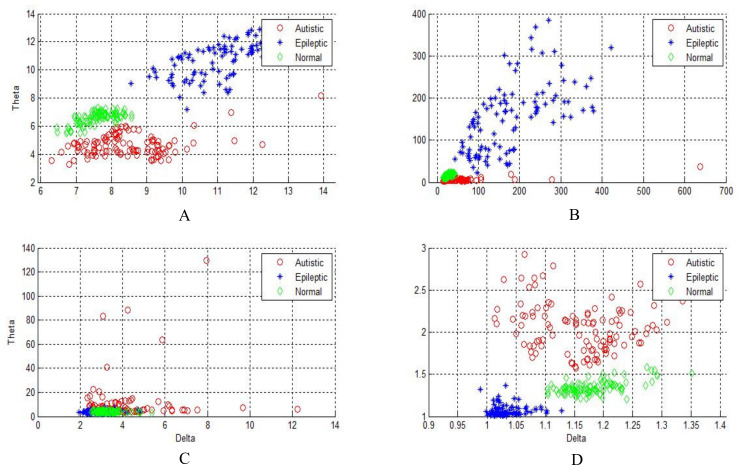
Two-dimensional plot of feature vectors of autistic, epileptic, and neurotypical data using (**A**) DWT + LBP, (**B**) DWT + standard deviation, (**C**) DWT + kurtosis, and (**D**) DWT + entropy.

Figure 4, Figure 5 and Figure 6 show two-dimensional plots of feature vectors extracted by LBP, SD, kurtosis, and entropy. The plots of the features extracted from the autistic with neurotypical, epileptic with neurotypical, and autistic and epileptic with neurotypical datasets are shown in Figure 4, Figure 5 and Figure 6, respectively. Delta and theta bands are selected and plotted to show the scattering of EEG signal features and show the features that will be classified easily and correctly. It is clear from the figures that the features extracted by DWT with entropy and LBP can be better separated than those extracted by other approaches. After the features are extracted, the LDA, SVM, KNN, and ANN techniques are applied to classify the extracted features, and the results are compared. In this part, the classification accuracies have been computed for epilepsy and autism compared to neurotypical subjects (two class) based on DWT with different feature-extraction methods and four types of classifiers as follows.

#### 3.1.1. Two-Class Neurotypical vs. Epilepsy (Single-Channel)

For single-channel EEGs of epilepsy, we used a dataset provided by Bonn University where 100 segments from set A for neurotypical and 100 segments from set E for epileptic were considered. Table 1 shows the classification accuracies of neurotypical and epilepsy for single-channel EEG signals based on our approaches. From Table 1, it can be seen that the combination of DWT with LBP and entropy are better than other combinations for single-channel EEG data and that the KNN classifier is a good classifier to achieve the highest classification accuracies.

By comparing our results in this section with other studies, we find that our work produced a better classification accuracy values than those reported in Nigam and grape [3] study that used Non-linear filter with ANN classifier with accuracy value 97.2%. Our study provides classification accuracy values 99.5 ± 0.5 using DWT + LBP and entropy with all classifiers, but DWT +entropy with KNN classifier produced accuracy value 98.5 ± 0.5%. Further, we find that our study provides the classification accuracy values better than the study of Kannathal et al. [4] that utilized entropies and ANFIS classifier with accuracy value 92.2%. Our approaches produced the classification accuracy values better than Sadati et al. [5] study. Sadati et al. [5] used DWT with ANFN classifier to achieve 85.9%. This study achieved classification accuracy values higher than the classification accuracy reported in Ocak [6], which utilized approximation entropy and ANN to achieve 96% classification accuracy.

However, studies by Chen [10] and Djemili et al. [11] achieved classification accuracy values equal to the values reported in this study. Chen [10] utilized dual-tree complex wavelet transform (DTCWT) with KNN classifier while Djemili et al. [11] used empirical mode decomposition (EMD) and multilayer perceptron neural network (MLPNN) classifier. 

The results achieved in this paper can be compared in more detail with the other studies listed in Table 3. Our study produced better results than similar studies reported in the literatures.

#### 3.1.2. Two-Class Neurotypical vs. Epilepsy (Multi-Channel)

For multi-channel EEGs of epilepsy, we used a dataset provided by CHB-MIT with 23 channels. The utilized dataset comprised 11 neurotypical subjects and 12 epileptic subjects. Table 2 shows the classification accuracies of neurotypical and epileptic subjects for multi-channel EEG signals based on our approaches. From Table 2, it can be seen that a combination of DWT with LBP and entropy are better than other combinations for multi-channel EEG data and that the SVM classifier is a good choice to achieve the highest classification accuracies. In general, for neurotypical and epilepsy diagnosis, single-channel EEG signals provide better accuracy than multi-channel EEG signals. Overall, the combination of DWT + (Entropy or LBP) + KNN and DWT + (Entropy or LBP) + SVM achieved the highest accuracies for single-channel and multi-channel EEG signals, respectively.

By comparing our results with other studies, we find that our study achieved the classification accuracy up to 99% higher than the other studies. Khan et al. [39] used CHB-MIT dataset and utilized DWT with LDA classifier to achieve classification accuracy up to 91.8%, and Subasi [40] used DWT and dynamic fuzzy neural network (DFNN) classifier to produce a classification accuracy value of 93.1%. Further, our study produced classification accuracy higher than Yuan et al. [40]. Yuan et al. [41] used the fractal intercept derived from fractal geometry as a nonlinear feature and the relative fluctuation as a linear feature and used Extreme learning machine (ELM) algorithm as a classifier to achieve a classification accuracy of up to 94.9%. The results achieved in this paper can be compared in more detail with the other studies listed in Table 3. Our study produced better results than similar studies.

#### 3.1.3. Two-Class Neurotypical vs. Autism (Single-Channel)

In this section the classification accuracy results of the proposed methods for autism diagnosis will be presented and discussed for two-class single channel mode. For single-channel EEGs of autism disorders, we use a dataset from King Abdulaziz University. The dataset comprises 10 neurotypical subjects and 9 autistic subjects. 

Table 4 shows the classification accuracies of neurotypical and autism for single-channel EEG signals based on our approaches. From Table 4, it can be seen that a combination of DWT with LBP and entropy is better than other combinations for single-channel EEG data and that the ANN and KNN classifiers can achieve the highest classification accuracies.

#### 3.1.4. Two-Class Neurotypical vs. Autism (Multi-Channel)

In this section, the classification accuracy results of the proposed methods for autism diagnosis will be presented and discussed for two-class multi-channel mode. The King Abdulaziz University dataset is used for multi-channel autism diagnosis. Table 5 shows the classification accuracies of neurotypical and autistic subjects for multi-channel EEG signals based on our approaches. From Table 5, it can be seen that a combination of DWT with LBP and entropy is better than other combinations for multi-channel EEG data and that the ANN and KNN classifiers can achieve the highest classification accuracies. In general, for neurotypical and autism diagnosis, multi-channel EEG signals provide better accuracy than single-channel EEG signals. Overall, the combination of DWT + (Entropy or LBP) + ANN achieved the highest accuracies for both single-channel and multi-channel signals.

By comparing our results in this section with other studies, we find that our work provides better classification accuracy values than those reported in other studies. Sheikhani et al. [13] used the short-time Fourier transform (STFT) and then used k-nearest neighbors (KNN) as a classifier to achieve classification accuracy up to 82.4%. In a later study [14], they developed the proposed method to obtain an accuracy of 96.4%. Ahmadlou et al. [16] discussed a visibility graph (VG) technique with wavelet and an enhanced probabilistic neural network classifier (EPNN) to obtain an accuracy of around 95.5%. Bosl [18] used the minimum mean-square error and KNN, naive Bayesian, and support vector machine (SVM) to produce classification accuracy ranged between 70% and 100%. A study by Alhaddad et al. [19] used time and frequency domains (raw data and Fast Fourier Transform (FFT)) and a fisher linear discriminant as a classifier to provide an overall classification accuracy up to 90%. The results achieved in this paper can be compared in more detail with the other studies listed in Table 6. Our study produced better results than similar studies reported in the literature.

### 3.2. Three-Class Classification

Finally, the three-class classification has been implemented. Epilepsy, ASD, and neurotypical data have been collected and presented. In our work, three-class diagnosis with single-channel and multi-channel mode have been studied. In our proposed system, several combinations of feature-extraction methods with several classification methods have been applied to achieve good accuracy. The classification accuracies have been computed for epileptic, autistic, and neurotypical individuals based on DWT with different feature-extraction methods and four types of classifiers.

#### 3.2.1. Three-Class Neurotypical vs. Epilepsy vs. Autism (Single-Channel)

For single-channel three-class diagnosis, we used a dataset from Bonn university comprising of 100 segments from set A for neurotypical and 100 segments from set E for epileptic and autistic and a dataset of 9 subjects provided by the King Abdulaziz University. 

Table 7 shows the classification accuracies of neurotypical, epilepsy, and autism for single-channel EEG signals based on our approaches. From Table 7, it can be seen that a combination of DWT with LBP and entropy is better suited than other combinations for single-channel EEG data and that all classifiers work well with the previous combinations to achieve the highest classification accuracies.

#### 3.2.2. Three-Class Neurotypical vs. Epilepsy vs. Autism (Multi-Channel)

For multi-channel three-class diagnosis, we used a dataset provided by MIT-USA with 23 channels. The dataset comprises 11 neurotypical subjects and 12 epileptic subjects. In addition, a dataset of 9 subjects was provided by King Abdulaziz University. Table 8 shows the classification accuracies of neurotypical, epilepsy, and autism data from multi-channel EEG signals based on our approaches. From Table 8, it can be seen that a combination of DWT with LBP and entropy is better suited than other combinations for multi-channel EEG data and that all classifiers work well with the previous combinations to achieve the highest classification accuracies.

By observing the results in two-class diagnosis (epilepsy versus Neurotypical and ASD versus Neurotypical), the methods proposed in this study show better performance and higher accuracies than those results in some previous studies. Besides, the results related to three-class diagnosis also show a high accuracy reaching up to 99% in some approaches. These results confirm the rigidity of the proposed methods despite the increase in the number of classes. 

Finally, we know most brain disorder diagnoses are performed manually by neurologists or skilled clinicians through visual inspection of EEG signals. Therefore, the proposed system can assist medical doctors and clinicians in order to diagnose neurological brain disorder automatically. With the proposed system, the number of neurologists that is limited can be reduced and diagnosis time is saved. The human brain is the most complex part of the human body and provides a wide variety of information related to neurological disorders. However, related to our study results, the proposed system can diagnose neurological brain disorders easily, successfully, and accurately.

Regarding the overfitting problem of the utilized intelligent classifiers, the research team tried to prevent this problem by several ways. K-fold cross validation technique was used in order to estimate the accuracy of classifier model and prevent the overfitting problem. It allows to train and test the classifier model k-times on different subsets of training data and build up to estimate the performance of the classifier model on unknown data. Then, the intelligent classifiers are trained with the 90% of EEG features because the training of classifiers with more features reduces the chances of overfitting. Next, the capacity of the classifier network is reduced in order to reduce the network complexity and avoid the overfitting problem. For example, one input, one hidden, and one output layer are selected, and the number of neurons in the hidden layer is reduced in ANN classifier. Finally, the early stopping process is investigated to measure classifier performance and stop the training of the classifier before passing the stopping point in order to prevent the overfitting problem. For example, the maximum number of iterations allowed in the SVM classifier is selected, and the maximum number of epochs, neural network gradient, mu, and performance parameters in ANN classifier are chosen carefully in order to satisfy the early stopping process and prevent the overfitting problem.

## 4. Conclusions and Future Study

EEG signal-analysis techniques have been improved in recent years because the EEG reflects neurological brain activity and is an important tool for diagnosing neurological brain disorders, such as autism, epilepsy, and Alzheimer’s disease. In this study, we develop a single system that diagnoses neurological diseases: epilepsy vs. Neurotypical, ASD vs. Neurotypical, and epilepsy vs. ASD vs. Neurotypical. In order to increase the diagnosis accuracy in the developed system, we investigate different techniques for EEG feature extraction and classification for single-channel mode and multi-channel mode EEGs. The different datasets used in this work are provided by Bonn University, Germany; MIT, USA; and King Abdulaziz University (KAU), Jeddah, Saudi Arabia, to evaluate our proposed design. The ICA technique is used to remove the eye blinks artifacts from EEG dataset. Then, the EEG data are segmented and filtered to remove the noise and interference, and then the EEG features are extracted from the filtered signal by DWT to decompose the filtered signal into its sub-bands. Five statistical methods are investigated to extract the features from the EEG sub-bands. The features are used as the inputs to four different classifiers to categorize the features into their corresponding classes. The combination of DWT with SE and LBP produces the highest accuracy for all classifiers. For epilepsy diagnosis, the classification accuracy approaches 100% with all classifiers and 99% with KNN for the two-class single-channel and multi-channel EEGs, respectively. For autism diagnosis, the classification accuracy reaches up to 91.5% and 99% with ANN for two-class single-channel and multi-channel modes, respectively.

The results of our proposed methods, whether two-class diagnosis and three-class diagnosis, show better performance and higher accuracies than those results in some previous studies. The overall classification accuracy approaches 99.9% with SVM and 97% with ANN for the three-class single-channel and multi-channel data, respectively. These results confirm the rigidity of the proposed methods despite the increase in the number of classes. In general, to distinguish neurotypical, epileptic, and autistic patients, single-channel EEG signals provide better accuracy than multi-channel EEG signals. Overall, the combination of DWT + (Entropy or LBP) + SVM and DWT + (Entropy or LBP) + ANN achieved the highest accuracies for single-channel and multi-channel data, respectively. In future work, we will test and evaluate our proposed methods with other datasets. Another neurological disorder will be included in our study. Our own dataset will be recorded in our lab for several types of neurological brain disorders.

## Figures and Tables

**Table 1:** Classification accuracy for neurotypical vs. epilepsy (single-channel).

Feature Extraction	Classification Accuracy (%)			
	LDA	SVM	KNN	ANN
DWT + LBP	99.5 ± 0.5	99.5 ± 0.5	99.5 ± 0.5	99.5 ± 0.5
DWT + SD	90 ± 1	99 ± 1	99 ± 1	95 ± 2
DWT + Variance	80 ± 2	92 ± 1	99 ± 1	66 ± 4
DWT + Kurtosis	90 ± 2	96 ± 1	95 ± 2	95 ± 2
DWT + Entropy	99.5 ± 0.5	99.5 ± 0.5	98.5 ± 0.5	99.5 ± 0.5

**Table 2:** Classification accuracy for neurotypical vs. epilepsy (multi-channel).

Feature Extraction	Classification Accuracy (%)			
	LDA	SVM	KNN	ANN
DWT + LBP	94 ± 1	98 ± 0.5	98.6 ± 0.5	98.6 ± 0.5
DWT + SD	88 ± 2	96.7 ± 0.5	96 ± 0.5	69 ± 3
DWT + Variance	81 ± 2	95.5 ± 0.5	93.5 ± 0.5	63.5 ± 2.5
DWT + Kurtosis	68.5 ± 0.5	72 ± 3	72 ± 1	73.5 ± 1
DWT + Entropy	88.5 ± 3	97.5 ± 1	91.5 ± 0.5	95.5 ± 0.5

**Table 3:** Epilepsy diagnosis studies and classification results.

Authors	Feature Extraction	Classifier	Dataset	Accuracy
Nigam and grape [3]	Non-linear filter	ANN	Bonn university	97.2
Kannathal et al. [4]	Entropies	ANFIS	Bonn university	92.2
Sadati et al. [5]	DWT	SNFN	Bonn university	86
Ocak [6]	Approximation entropy +DWT	ANN	Bonn university	96
Nunes et al. [7]	wavelet	Optimum path forest	Bonn university	89.2
Subasi et al. [8]	DWT	PCA-LDA ICA-SVM	Bonn university	98–100
Subasi [9]	DWT	Mixture of expert model	Bonn university	94.5
Chen [10]	DTCWT	KNN	Bonn university	100
Khan et al. [39]	DWT	LDA	CHB-MIT	91.8
subasi [40]	DWT	DFNN	Own dataset	93.1
Yuan et al. [41]	fractal intercept and relative fluctuation	ELM	Own dataset	94.9
Patel et al. [42]	------	SVM-LDA QDA-MDA	Own dataset	76.5–87.7
Bao et al. [43]	------	PNN	Own dataset	94.07

**Table 4:** Classification accuracy for neurotypical vs. autism (single-channel).

Feature Extraction	Classification Accuracy (%)			
	LDA	SVM	KNN	ANN
DWT + LBP	84 ± 0.5	85.2 ± 0.4	90.4 ± 0.3	91.2 ± 0.3
DWT + SD	74.4 ± 0.5	82.7 ± 0.2	88 ± 0.5	89 ± 1
DWT + Variance	49 ± 1	75.5 ± 0.5	85.3 ± 0.5	72 ± 4
DWT + Kurtosis	63.8 ± 0.5	58.4 ± 0.4	79.2 ± 0.4	78 ± 1
DWT + Entropy	86.2 ± 0.2	86 ± 0.2	90.5 ± 0.3	90.8 ± 0.2

**Table 5:** Classification accuracy for neurotypical vs. autistic (multi-channel).

Feature Extraction	Classification Accuracy (%)			
	LDA	SVM	KNN	ANN
DWT + LBP	95.3 ± 0.5	96.5 ± 0.5	95.2 ± 0.5	97.1 ± 0.5
DWT + SD	89 ± 1	92 ± 1	91 ± 1	94 ± 1
DWT + Variance	83 ± 1	83 ± 1	90 ± 1	67 ± 3
DWT + Kurtosis	73 ± 1	78 ± 2	82 ± 1	78 ± 2
DWT + Entropy	97.5 ± 0.5	97.6 ± 0.5	97.9 ± 0.5	98.2 ± 1

**Table 6:** Autism diagnosis studies and classification results.

Authors	Feature Extraction	Classifier	Dataset	Accuracy
Sheikhani et al. [13]	STFT	KNN	Own dataset	82.4
Sheikhani et al. [14]	STFT and statistical	KNN	Own dataset	96.4
Ahmadlou et al. [15]	Wavelet and fractal dimension	RBNN	Iranian dataset	90
Ahmadlou et al. [16]	Wavelet and visibility graph	EPNN	Iranian dataset	95.5
Ahmadlou et al. [17]	Wavelet and fuzzy logic	EPNN	Iranian dataset	95.5
Bols et al. [18]	Modified multiscale	SVM	Own dataset	70–100
Alhaddad e al [19]	FFT	FLDA	Own dataset	90
Alsaggaf et al. [20]	FFT	FLDA	Own dataset	80.27
Fan et al. [21]	FFT	BN, MLP, NB, SVM, RF, KNN, j48	Own dataset	75–85

**Table 7:** Classification accuracy for neurotypical vs. epilepsy vs. autism (single-channel).

Feature Extraction	Classification Accuracy (%)			
	LDA	SVM	KNN	ANN
DWT + LBP	99 ± 0.05	99.7 ± 0.1	99 ± 0.05	98.3766
DWT + SD	77 ± 1	96.9 ± 0.3	98.3 ± 0.3	96.2 ± 1
DWT + Variance	60.7 ± 0.5	65.5 ± 0.5	96.9 ± 0.3	73.3 ± 0.3
DWT + Kurtosis	58.3 ± 0.3	67.5 ± 0.5	84.7 ± 0.7	84.4156
DWT + Entropy	97.9 ± 0.3	99.9 ± 0.1	98.4 ± 0.1	98.7 ± 1

**Table 8:** Classification accuracy for neurotypical vs. epilepsy vs. autism (multi-channel).

Feature Extraction	Classification Accuracy (%)			
	LDA	SVM	KNN	ANN
DWT + LBP	96.6 ± 0.3	96.7 ± 0.5	95.8 ± 0.5	97 ± 1
DWT + SD	91.5 ± 0.2	92.1 ± 0.1	93.6 ± 0.3	73.7 ± 1
DWT + Variance	73.8 ± 2.5	67.5 ± 0.6	88.3 ± 0.6	61.6 ± 0.6
DWT + Kurtosis	63.8 ± 0.2	65.2 ± 0.7	65.9 ± 0.5	65.5 ± 2.5
DWT + Entropy	95.2 ± 0.2	95.5 ± 0.2	93.9 ± 0.5	96.2 ± 1
